# An Overview of Essential Nutritional Strategies and Products in the Treatment of Endometriosis

**DOI:** 10.3390/nu18010077

**Published:** 2025-12-26

**Authors:** Małgorzata Szczuko, Maciej Ziętek, Katarzyna Janda-Milczarek, Ewa Rębacz-Maron, Jolanta Nawrocka-Rutkowska, Kamila Pokorska-Niewiada

**Affiliations:** 1 Department of Bromatology and Nutritional Diagnostics, Pomeranian Medical University in Szczecin, 70-111 Szczecin, Poland; 2 Department of General Pharmacology and Pharmacoeconomics, Pomeranian Medical University in Szczecin, 71-460 Szczecin, Poland; maciej.zietek@pum.edu.pl; 3 Medical Biotechnology and Laboratory Medicine, Department of Biology, Parasitology and Pharmaceutical Botany, Faculty of Pharmacy, Pomeranian Medical University in Szczecin, 70-111 Szczecin, Poland; katarzyna.janda@pum.edu.pl; 4 Department of Ecology and Anthropology, Institute of Biology, University of Szczecin, Wąska 13, 71-415 Szczecin, Poland; ewa.rebacz-maron@usz.edu.pl; 5 Department of Gynecology, Endocrinology and Gynecological Oncology, Pomeranian Medical University in Szczecin, 71-252 Szczecin, Poland; jolanta.nawrocka@pum.edu.pl; 6 Department of Toxicology, Dairy Technology and Food Storage, West Pomeranian University of Technology in Szczecin, 71-454 Szczecin, Poland

**Keywords:** endometriosis, diet therapy, nutrition, elimination, allergies, inflammation

## Abstract

Background/Objectives: Recent reports on the co-occurrence of allergies and endometriosis have provided grounds for expanding research in this area, suggesting that diagnostics should be extended to women with endometriosis. However, numerous studies on nutrients and antioxidants do not specify the type of diet that supports the treatment process. In our review, we focus on the types of food elimination and dietary approaches that have been used. Methods: This systematic review was conducted according to the PRISMA guidelines. We searched the EMBASE, PUBMED and SCOPUS databases, as well as the bibliographies of research papers and reviews, including the latest reports from June 2025. The search keywords were “endometriosis” and “type of diet”, “nutrition”, “food products”, “nutrients”, “elimination diet”, and “allergies”. Results: Excluding coexisting allergies and introducing an anti-inflammatory diet low in animal products, limiting butter and margarine, and eliminating fried foods and refined simple sugars may be the best solution to help treat endometriosis. Conclusions: Personalised nutritional counselling for patients with endometriosis is particularly helpful and necessary, as there is no single elimination diet that can be recommended for all patients with endometriosis. The first step should be an anti-inflammatory diet, such as the Mediterranean diet or the MIND diet (Mediterranean-DASH diet intervention for neurological delay), followed by more in-depth allergy screening. The phenotypic diversity of this group of patients may require the use of a low-FODMAP (fermentable oligo-, di-, monosaccharides and polyols), low-nickel, gluten-free or other elimination diet.

## 1. Introduction

Recently, there has been increased interest in studying the relationship between endometriosis and dietary components. Although many systematic reviews and analyses have been published, they do not establish a cause-and-effect relationship, nor do they contain any recommendations for selecting or considering a specific diet.

The latest reports suggest, among other things, increasing the proportion of fruit and vegetables in the daily diet, avoiding red meat, and limiting gluten intake. These recommendations are primarily based on observations of a reduction in endometriosis-related pain symptoms after a change in diet. However, it has not yet been conclusively proven that this reduction in symptoms is caused by dietary changes. Studies do not take into account factors such as interactions between individual nutrients. A single food ingredient may have different properties (increased or decreased) than the same ingredient in a given diet. A good example of this is the difference between taking omega-3 fatty acid supplements and eating fatty fish, which contain large amounts of these fatty acids [1,2].

Symptoms associated with coexisting gastrointestinal disorders, to which women with endometriosis are particularly susceptible, were also not considered. These include irritable bowel syndrome (IBS), Crohn’s disease, and ulcerative colitis [3,4,5]. Despite the high prevalence of gastrointestinal problems, effective treatments are lacking. While pharmacotherapy can relieve pain and menstrual symptoms associated with endometriosis, it has no effect on gastrointestinal symptoms and may even exacerbate them [6]. In 1996, the American Society for Reproductive Medicine (ASRM) introduced a classification of endometriosis based on the size and location of lesions [5].

- First-degree endometriosis (minimal)—small foci of endometriosis are located on the uterus, ovaries, fallopian tubes, and peritoneum, and there may be small adhesions.
- Second-degree of endometriosis (mild)—more extensive foci of endometriosis appear, and endometrial cysts of the ovaries (also known as chocolate cysts) may be present; changes may be located behind the uterus, in the rectouterine pouch.
- Third-degree endometriosis (moderate)—extensive adhesions are present, and the disease process also affects the sacrouterine ligaments.
- Fourth-degree endometriosis (severe)—due to the presence of numerous adhesions, deformation of the surrounding organs occurs. For example, the uterus may be immobile, bent backwards, or attached to the intestinal loops. Endometriosis lesions may also occur in other organs, including the appendix, bladder, intestines, vagina, and cervix.

There is very little scientific evidence supporting a dietary approach to endometriosis, and most of this concerns dietary supplements with antioxidant, antiproliferative, anti-inflammatory and anti-angiogenic properties [4]. Recent interesting reports on the co-occurrence of allergies in endometriosis have provided a basis for expanding research in this area. Therefore, we have decided to conduct a review to address this gap in the literature. As patients with endometriosis exhibit diverse phenotypes and the symptoms are non-specific, our review aims to recommend appropriate diets depending on symptom severity and endometriosis stage. We assumed that eliminating specific foods from the diet of all women with endometriosis might not be significant. The selection of an elimination diet should be personalized, and we are interested in such studies.

## 2. Materials and Methods

In order to ensure effective methodological integrity, validity and quality, the PRISMA guidelines were used. For the purposes of this systematic review, original research papers found in the EMBASE, PUBMED, and SCOPUS databases were selected. Each database search was conducted independently, and selected articles published by the end of June 2025 were collated, but not older than 20 years.

We used the following keywords in the search process: “endometriosis” and “type of diet”, “nutrition”, “food products”, “nutrients”, “elimination diet”, and “allergies”. Relevant studies and review papers examining the relationship between EM and the selected factors were included, with eligibility limited to publications written in English. In order to avoid the possibility of important sources being overlooked, a manual search was also conducted of the reference lists of selected articles. Furthermore, experts in the field were consulted for their opinion on key publications.

**Figure 1:**
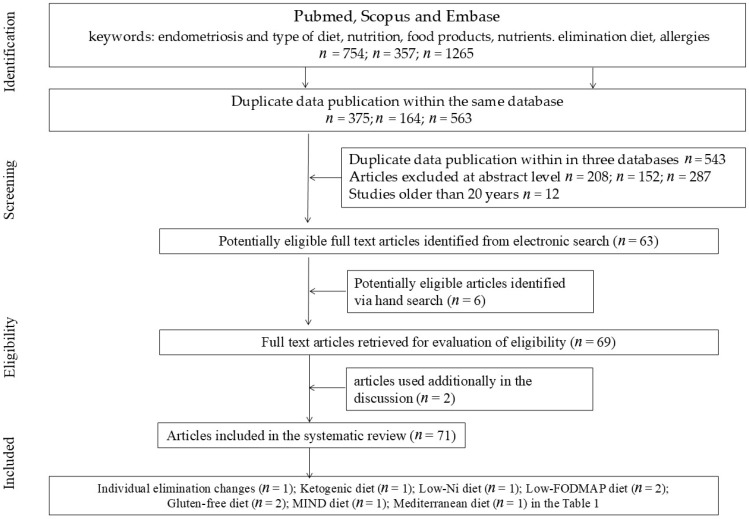
PRISMA Flow diagram.

The inclusion criteria comprised original research articles, biochemical analyses, the presence of a control group, publication in English, and appearance in peer-reviewed scientific journals. The exclusion criteria encompassed animal studies, publications that had been retracted, articles lacking open access, and papers with incomplete or insufficient data. The literature search was conducted independently by three authors, who also removed duplicate records identified across the three databases. In order to ensure rigorous methodology and minimize bias, articles were selected according to a multi-stage procedure (Figure 1).

This strategy ensured that all relevant, high-quality data aligned with the study’s objectives were incorporated. Table 1 presents the analysed studies. Research pertaining to the consumption of red meat and dairy was excluded from consideration due to the intricacies inherent in these subjects, which extend beyond the scope of specific product consumption, as elaborated in the subsequent subsections.

## 3. High-Fat and/or High-Protein Diets

The component of the diet that has been most subject to controversy is fat. It is a source of energy and nutrients, and bioactive fatty acids can affect cellular metabolism, inflammation, and endogenous estrogen levels [7,8]. This group is characterized by a high degree of diversity, encompassing saturated fatty acids (SFA), monounsaturated fatty acids (MUFA), and polyunsaturated fatty acids (PUFA), including omega-3 and omega-6. The latter appear to be of particular significance due to their anti-inflammatory (omega-3) and pro-inflammatory (omega-6) properties [9,10,11].

The ketogenic diet is the most popular fat-focused dietary regime. The diet is characterized by a high fat intake, with a concomitant restriction of carbohydrate consumption. The predominant fat type in this dietary regime is animal fat, which is characterized by a low content of unsaturated fatty acids. Furthermore, the restriction of low-molecular-weight antioxidants and the diminution of antioxidant enzymes (due to constrained protein intake) can result in the onset of diseases associated with oxidative stress. However, contradictory studies have been published, which suggest that caloric restriction can enhance antioxidant levels [12,13]. However, studies conducted on patients with endometriosis have shown only a marginal, significant reduction in the final scores of pelvic pain assessment Table 1 [14].

As demonstrated in the relevant animal studies, a high-fat diet has been shown to exacerbate endometriosis by increasing systemic inflammation and oxidative stress. This suggests that consuming large amounts of fat has a negative impact [7].

### 3.1. Red Meat

The effectiveness of dietary interventions in women with endometriosis is a contentious issue, with contradictory reports including those concerning the consumption of meat and cold cuts [15,16]. A number of reports have indicated a correlation between the consumption of substantial quantities of beef and other red meats and an elevated risk of developing endometriosis in women, suggesting a potential pro-inflammatory effect of such diets [17,18]. However, other authors [19,20] do not confirm these reports.

It is important to consider the potential impact of meat consumption on the progression of endometriosis, given the established relationship between estrogen levels and the development of this condition. The presence of small amounts of estrogen in meat products may contribute to the development of endometriosis, highlighting the need for further research in this area. Evidence has been presented which indicates that the consumption of red meat should be limited due to the potential for bacterial or microbial contamination and dysbiosis following its ingestion [1,21,22]. The potential implications of dysbiosis in endometriosis may be explained by the following factors: immune system activation, cytokine-mediated intestinal dysfunction, impaired oestrogen metabolism and signalling, and impaired progenitor and stem cell homeostasis [22]. Another potential cause of the observed associations (apart from the influence of steroid hormones contained in meat and microflora) is the theory that heme iron contained in meat, acting as a prooxidant, may stimulate inflammation [18]. As Trabert et al. [19] observed, the potential impact of heme iron may be realised with a weekly consumption of a minimum of seven servings of red meat.

A comprehensive analysis of potential sources of error that could distort the results is therefore required to provide a robust conclusion. In addition, anthropometric indicators, especially BMI (body mass index), should be taken into account. The consumption of substantial quantities of meat has been demonstrated to be a contributing factor to the prevalence of obesity [23]. Conversely, being overweight has been demonstrated to induce chronic low-grade inflammation [24]. In the context of analyses exploring the relationship between meat consumption and inflammation, the consideration of overweight individuals has been identified as a potential marginalising factor [25].

As demonstrated in studies [26,27,28], obesity has been shown to be associated with elevated levels of estradiol in both serum and adipose tissue. It can be hypothesized that the incorporation of BMI into the analysis would result in a divergence of conclusions among certain authors [18,29]. Furthermore, the hypothesis that steroid residues in meat could play a significant role in the aetiology of endometriosis appears to be unconvincing [29].

In summary, the available literature does not confirm a causal relationship between red meat consumption and the risk of endometriosis. There is a lack of typical clinical studies. Conclusions regarding the reduction of red meat consumption are based solely on survey studies Furthermore, none of the aforementioned studies incorporated the potential for systematic error in the results, which may have arisen from the morphometric characteristics of the patients.

### 3.2. Dairy Products

The hypothesis that the consumption of dairy products, which contain significant quantities of vitamin D, calcium, and magnesium, may result in a reduced risk of endometriosis is supported by evidence derived from studies of the subject’s relationship to nutritional intake [16,19,29,30,31,32]. The analysis revealed that women who consumed more than three servings of dairy products daily exhibited a 18% reduced probability of receiving a diagnosis of endometriosis, in comparison with those who consumed two servings daily [31].

Some reports even indicate that dairy products have a pro-inflammatory effect, which is explained by the presence of small amounts of estrogen in cow’s milk and the stimulation of insulin-like growth factor 1 (IGF-1) [33]. Activation of insulin/IGF-1 receptors has been shown to stimulate STAT3 signal-ling. This, in turn, triggers a negative feedback reaction, resulting in insulin resistance and activation of immunosuppressive cells during the aging process. Its excess promotes the formation of pro-inflammatory cytokines, which can not only exacerbate inflammation, but also endometrial pain [34,35,36]. However, the content of vitamin D and calcium in skim dairy products has been demonstrated to balance or even exceed these processes. Harris et al. hypothesized that a slight increase in daily dairy intake (one additional serving per day) could result in up to a 5% reduction in the risk of endometriosis [30]. Consequently, it is crucial to implement a suitable dietary regime grounded in scientific evidence.

## 4. A Diet Low in Fermentable Oligo-, Di-, Monosaccharides and Polyols (Low-FODMAP Diet)

The low-FODMAP diet is intended for people with visceral hypersensitivity, such as irritable bowel syndrome (IBS) [37]. The idea of using the low-FODMAP diet in endometriosis stems from similar gastrointestinal symptoms. Furthermore, approximately 20% of women diagnosed with endometriosis also exhibit symptoms consistent with IBS, and women with IBS report an increase in pain during menstruation in over 50% of cases. The dietary modification strategy, encompassing the restriction of short-chain fermentable carbohydrates, has been demonstrated to result in a substantial reduction in visceral symptoms among patients diagnosed with IBS. It is hypothesized that intestinal dysbiosis may be responsible for this problem, which is also significant in patients with endometriosis [37,38,39,40]. The results of the low FODMAP diet in women with endometriosis are presented in Table 1. Following the implementation of the intervention, a reduction in pathophysiological changes and intestinal symptoms was observed [4,39].

The reduction in intestinal symptoms observed in patients diagnosed with endometriosis and IBS following the implementation of the low-FODMAP diet suggests the potential for shared pathogenic pathways [40]. It is unfortunate that thus far, these have not been explained.

## 5. Gluten-Free Diet

It has been proven that a diet rich in gluten (a mixture of proteins, mainly gliadin and glutenin, found in many types of grains) can cause inflammatory reactions in some people. The use of a gluten-free diet by patients with endometriosis, similar to a low-FODMAP diet, stems from the desire to reduce digestive discomfort. This type of dietary modification has been shown to relieve pelvic pain and gastrointestinal symptoms [41,42]. This may be due to the reduction in gliadin proteins in the diet, whose ability to increase mast cell levels can stimulate an inflammatory response. Eliminating this factor may alleviate inflammation. This may explain the reduction in pain symptoms in patients with endometriosis who have eliminated gluten from their diet. What is more, after stopping the gluten restriction, symptoms such as pain, bloating, diarrhea/constipation returned [43,44].

Modifying the diet of women with endometriosis by eliminating gluten and dairy products while increasing the amount of vegetables and fiber consumed significantly reduced pain (Table 1) [45,46]. However, a more detailed analysis of the improvement in the quality of life of these patients indicated that this was the result of the combined properties of individual nutrients. Separate analyses of each food, as well as each nutrient, showed no significant changes in pain perception. Furthermore, dietary interventions did not demonstrate efficacy in mitigating this symptom. A gluten-free diet brings relief from intestinal discomfort to many people, but it can result in many negative reactions, such as nutritional deficiencies, intestinal microbiome disorders, or a deterioration in the well-being of patients. This is due to the need to find gluten-free equivalents of previously used food products, which are more difficult to obtain and often more expensive than regular ones. Furthermore, it is imperative that patients acclimatise to a new sensory experience. This does not improve their quality of life [4].

Patients diagnosed with endometriosis and exhibiting severe intestinal symptoms subsequent to gluten and/or wheat consumption should undergo gastroscopy in order to ascertain the presence of coexisting celiac disease and/or non-celiac gluten sensitivity. This procedure is undertaken with a view to excluding the potential influence of gluten on symptoms related to endometriosis. Consequently, the recommendation of a gluten-free diet for all patients diagnosed with endometriosis, as is observed in cases of Hashimoto’s disease, appears to be both futile and ill-advised [46,47].

Since the use of a gluten-free diet is potentially associated with a risk of low fiber intake, education and guidance should be provided to patients with endometriosis in order to select appropriate fiber-rich products.

## 6. Low-Nickel Diet

Nickel (Ni) is a trace element that is commonly found in the environment (air, water, soil) and in living organisms, including in the diet. The extent to which this process contributes to human health and well-being remains to be elucidated [48]. Nickel has been observed to be a contributing factor in the development of allergies, with the potential to induce allergic contact mucositis (ACM) when ingested orally. Estimates suggest that the prevalence of ACM is over 30%. The condition can present itself with symptoms similar to IBS. It is hypothesised that the increased prevalence of nickel in the blood of women diagnosed with endometriosis may be attributable to the potential of nickel to exert estrogenic effects [49].

It has also been observed that women with endometriosis are more likely to experience nickel allergies, although other allergic disorders have not been linked to the disease. A correlation has been observed between the occurrence of nickel allergy symptoms in women diagnosed with endometriosis and the manifestation of gastrointestinal symptoms. Foodstuffs with high levels of this element include dairy products, cereals, vegetables, legumes, nuts, and seeds (Table 1) [49,50]. The restriction of foods containing elevated levels of nickel in women diagnosed with endometriosis led to a substantial decrease in a range of gastrointestinal and gynecological symptoms, including those that are indicative of endometriosis, such as chronic pelvic pain, painful menstruation, and dyspareunia [49].

This is probably related to the higher percentage of women with contact allergy to nickel accompanying endometriosis. A nickel-free diet may be recommended for women with endometriosis who also have ACM [50].

## 7. Mediterranean Diet

The Mediterranean diet is characterized by high consumption of vegetables, fruits, nuts, seeds, legumes, and whole grains. The consumption of monounsaturated and polyunsaturated fatty acids, derived from fish, seafood, olive oil, avocados, nuts, and seeds, is also recommended. In addition, it is recommended to limit the consumption of eggs, red meat, and sweets [51].

Introducing changes in the diet of women with endometriosis, consisting of prohibiting the consumption of red meat, sweets, animal fats, and sweetened beverages, significantly reduced pain, dyspareunia, and dyshesion (Table 1) [52]. As demonstrated in other studies, a diet comprising high quantities of green vegetables, red meat, dairy products, fresh fruit and legumes has been shown to be significantly correlated with a lower risk of endometriosis [53,54].

The components of the Mediterranean diet, including olive oil, fruits and vegetables, grains, and herbs, are rich in antioxidants, polyphenols, and anti-inflammatory compounds, which are recommended for the treatment of endometriosis [55]. This diet is worth considering in the treatment of this disease, as it helps to inhibit the development of inflammation in the body and maintain hormonal balance. Unfortunately, to date, there are few studies that clearly confirm the health benefits of the Mediterranean diet in women with endometriosis.

## 8. MIND Diet (Mediterranean-DASH Diet Intervention for Neurological Delay)

The MIND diet is a diet that combines the Mediterranean diet to stop hypertension (DASH) and dietary interventions to delay neurodegenerative development.

It is a diet rich in whole grains, vegetables (especially leafy greens), fruits (especially berries), fish, poultry, and olive oil. It eliminates red meat, solid fats, cookies and sweets, and highly processed foods from the diet [56].

The introduction of the MIND diet in the case of endometriosis seems to be justified due to its anti-inflammatory properties and richness in vitamins, carotenoids, and flavonoids with proven health benefits. However, the results of the studies are contradictory.

Increasing the consumption of green leafy vegetables and other vegetables, nuts, legumes, berries, and fish in the diet significantly reduced the risk of endometriosis. Red meat, butter, and margarine, on the other hand, increased this risk. Unfortunately, whole grains may also have pro-inflammatory properties [57]. Nevertheless, there are few clinical studies confirming the effectiveness of this diet, so it should only be considered after consulting a doctor (Table 1) [58].

## 9. High-Fiber Diet

A high-fiber diet is one that contains both soluble and insoluble fiber in large amounts. It can include fruits, vegetables, whole grains, lentils, legumes, nuts, and seeds. Fiber helps remove estrogen from the body by forming bonds with it [59,60]. Lowering estrogen levels in women with endometriosis is important because it inhibits the spread of endometrial tissue. Dietary research and modifications for women with endometriosis focus largely on fruits and vegetables. It has been shown that increasing the proportion of citrus fruits in the daily diet significantly reduces the risk of endometriosis (by more than 20%) [59]. Increasing the amount of cruciferous vegetables consumed daily increases the risk of endometriosis by 13% [61]. Although other studies confirm the link between increased consumption of vegetables and fruit and a reduced risk of endometriosis, these studies, being case–control studies, may be subject to a large margin of error [61].

Research has demonstrated that women who consume higher amounts of fiber have lower levels of estradiol, the primary estrogen that is implicated in the development of endometriosis. The spread of endometrial tissue, as well as the chronic inflammation and pain that accompany it, are largely regulated by the hormones E2 and ERβ [62].

A plant-based diet, high fiber intake, and a diverse microbiome help increase the binding and excretion of sex hormones. A high-fiber diet has been shown to significantly reduce serum estrogen levels [63]. In addition, a high-fiber diet can reduce mast cell activation [64]. It has been shown that implementing a high-fiber diet, based on the consumption of large amounts of vegetables and fruits, both raw and fermented, increases the diversity of the microbiome, affecting the estrobolome, as well as reducing oxidative stress and inflammation [65]. However, it is important to be aware of the possible side effects of excessive fiber consumption, which may include flatulence. Unfortunately, there are no reports on the impact of this type of diet on the course of endometriosis.

## 10. Antioxidant (Anti-Inflammatory) Diet

An antioxidant diet is characterized by a high intake of foods with anti-inflammatory properties. An anti-inflammatory diet is distinguished by a diet comprising a variety of fruits and vegetables, which provide the body with significant quantities of polyphenols, anthocyanins, isoflavones, and lignans [45,66,67]. In addition, the consumption of fatty fish (e.g., salmon, trout, mackerel), whole grains, healthy fats (e.g., olive oil) and spices with anti-inflammatory properties (e.g., turmeric and ginger) is also recommended. In women diagnosed with endometriosis, the implementation of an anti-inflammatory dietary regime has been demonstrated to engender a substantial reduction in systemic inflammation, concomitant with a notable alleviation of the associated symptoms. This, in turn, has resulted in a marked enhancement in the quality of life experienced by patients [68,69,70]. Due to the positive effect of an antioxidant diet on endometriosis, it is recommended to expand research to include different stages and phenotypes of endometriosis. An anti-inflammatory diet may be beneficial not only for patients diagnosed with endometriosis, but also for other people.

**Table 1:** Research on the types of diets used in endometriosis treatment and their effects.

The Type of Elimination	Number of Subjects	Materials and Methods	Period	Effect	Authors	Research Project
Individual elimination changes	12 cases	Reducing consumption of dairy products and gluten Increasing consumption of fruit, vegetables, and fish	Minimum 1 year	Experience reduced pain and a regulated menstrual cycle	Vennberg et al., 2020 [2]	Quasi-experimental studies
Ketogenic diet with MCT supplement	19 cases/25 placebo	The modified MCT ketogenic diet contains 70–80% fat, 15–20% protein, and 5–10% carbohydrates.	12 weeks	dyspareunia and dyschezia significantly decreased marginally significant reduction in final pelvic pain scores	Naeini et al., 2025 [14]	Randomized Controlled Trial
Low-FODMAP diet	59 cases IBS/101 controls Endometriosis and IBS	Rome III criteria for IBS two groups: individuals with concurrent endometriosis and IBS, and individuals diagnosed solely with IBS.	4 weeks	improvement in intestinal symptoms in over 75% of patients	Moore et al., 2017 [38]	Randomized Controlled Trial
Low-FODMAP diet	50 cases/50 controls	7-day meal planning FODMAP intake < 5 g/day	28 days	Significant: improved stool consistency, improved overall quality of life, reduced intestinal gas production, relief of pain symptoms, relief of depression symptoms.	Varney et al., 2025 [40]	Randomized Controlled Trial
Gluten-free diet	207 patients	-	12 months	75%—statistically significant reduction in pain symptoms 25%—no improvement in symptoms 100%—significant improvement in quality of life	Marziali et al., 2012 [43]	Quasi-experimental studies
Gluten-free diet	204/114/56 controls	3 groups: 1–3 months; Less than 1 month Control group	1–3 months	Patients who avoided wheat, experienced greater improvements in well-being and reduced pain.	Mills et al., 2011 [46]	Randomized Controlled Trial
Low-Ni diet	31 cases	oral mucosal patch test Ni (omPT),	3 months	gastrointestinal, parenteral, and gynecological symptoms were statistically significantly reduced	Borghini et al., 2020 [50]	Quasi-experimental studies
Mediterranean diet	68	Doctors should recommend as a long-term solution for endometriosis	5 months	Significant reduction in pain and improvement in quality of life	Ott et al., 2012 [52]	Quasi-experimental studies
MIND diet	115 cases/230 controls	The MIND diet calculations were based on food intake in the form of FFQ.	8 months	A significant reduction in the risk of endometriosis was observed in the group consuming the most green leafy vegetables, other vegetables, nuts, legumes, berries, and fish. A higher risk of endometriosis was found in the group consuming the most red meat, butter, and margarine.	Noormohammadi et al., 2025 [58]	Randomized Controlled Trial

## 11. Discussion

Endometriosis is a disease involving the growth of endometrial tissue outside the uterine cavity. It is associated with fibrosis and inflammatory response. Endometriosis is considered one of the most painful female diseases [71]. The pain is associated with, among other things, menstruation, sexual intercourse, bowel movements, and digestive disorders. Factors that increase the risk of endometriosis include early menarche and short menstrual cycles [57]. There is no effective cure or preventive measures for this disease. Furthermore, there is no standard approach to treating these symptoms, and the disease has the potential to recur even after appropriate surgical or pharmacological interventions [72].

As indicated in the literature, dietary modifications in women with endometriosis can alleviate or limit some of the symptoms associated with the disease. Our literature review shows that it is not conclusive whether a single dietary and nutritional intervention is the most appropriate adjunctive therapy for endometriosis. A personalized approach to selecting the appropriate dietary and nutritional intervention, taking into account the comprehensive clinical history and lifestyle of the patient with endometriosis, may be useful. It is important to understand how food affects estrogen levels, the microbiome, and inflammation [73] to prevent the pathophysiological mechanisms in endometriosis.

**Figure 2:**
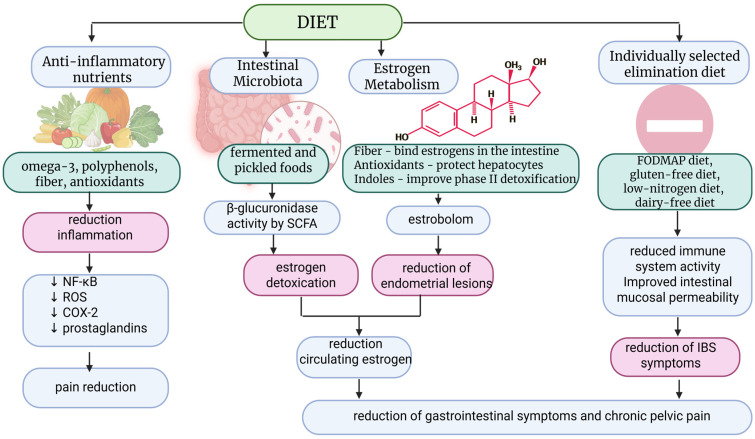
Mechanisms of dietary intervention in endometriosis. SCFA—Short-Chain Fatty Acids; ROS—Reactive oxygen species; NF-kB—Nuclear Factor kappa-light-chain-enhancer of activated B cells; COX-2—cyclooxygenase two; IBS—irritable bowel syndrome. Created in BioRender.com. Szczuko M. (2025) https://BioRender.com/gnudngb (accessed on 24 December 2025). ↓ indicate “reduce”.

It appears that this is a group of patients predisposed to allergies to various dietary and contact factors, which should be diagnosed. Several recognized risk factors for endometriosis also have a common denominator in the form of increased exposure to estrogens. Legumes, which are a source of phytoestrogens, may play an important role in lowering serum estrogen levels. In addition, plant-based diets, with their anti-inflammatory nature, have been found to be associated with increased levels of sex hormone-binding globulin, reducing the amount of bioavailable estrogen present in the body [68]. The nature of the disease, such as inflammation and estrogen activity, menstrual cycles, and the biochemistry of the arachidonic acid cascade, including the activation of the prostaglandin synthesis pathway, can influence dietary choices. Therefore, fatty fish containing eicosapentaenoic acid and docosahexaenoic acid (EPA and DHA), which are long-chain omega-3 fatty acids from which anti-inflammatory mediators such as meresins, protectins, and resolvins are formed, are an important part of an anti-inflammatory diet [74]. The MIND diet offers great hope. In addition to its similarities to the Mediterranean diet, it is more restrictive in terms of avoiding red meat, butter, and fried foods, which are products high in fat, simple sugars, and sweets, which have a pro-inflammatory effect [58]. It therefore appears that the MIND diet contains the necessary components to protect against the risk of endometriosis, as evidenced by the literature, and may even have an advantage over the Mediterranean diet in terms of reducing the consumption of meat and fat, including saturated and trans fatty acids, although such severe restrictions are probably not necessary [58]. The most promising interventions are the Mediterranean and MIND diets. Due to their health-promoting and anti-inflammatory nature, the use of these diets may also be important in the prevention of many diseases, including endometriosis. The nutritional requirements of patients diagnosed with endometriosis are subject to alteration in response to estrogen-progestogen therapy. The reduction in ovarian function decreases estrogen production, which inhibits inflammation, stabilizes the ectopic endometrium, modulates the gut microbiota and estrogen-related “estrobolom,” and alters liver and lipid metabolism [65,73]. These mechanisms have been demonstrated to exert a significant impact on the metabolism of vital nutrients including vitamins, minerals, fatty acids, and proteins. Therefore, the metabolism of folic acid, B vitamins, and homocysteine can lead to reduced levels of B6, B12, and folic acid [75]. In addition, elevated homocysteine levels, impaired methylation, and increased susceptibility to oxidative stress are observed. Nutritional recommendations include increased intake of green vegetables, legumes (folic acid), eggs, meat, dairy products (B12), whole grains, and bananas (B6) [75]. In cases where patients are prescribed oral contraceptives, it may be necessary for them to take supplements containing methylated forms of 5-MTHF, B6-P5P, and B12-methylcobalamin. Increased dietary intake of antioxidants such as vitamin C, vitamin E, and the polyphenols found in the Mediterranean diet effectively supports hormone therapy by reducing inflammation [53,54,55]. To simplify the understanding of dietary management in a group of patients with endometriosis, a shortened diagram of the principles of dietary intervention is presented below (Figure 2).

It appears that a combination of interventions, including personalised nutritional counselling, the exclusion of coexisting allergies, and the introduction of an anti-inflammatory diet low in animal products, butter and margarine, fried foods, and sweets, may represent the most efficacious approach for the management of endometriosis. The present review is subject to certain limitations, arising from the analysis of RCTs and observational studies, which may pose a higher risk of bias.

## 12. Conclusions

Various dietary approaches have been studied in women with endometriosis, showing promising effects in reducing pain and improving overall symptoms of the condition. Understanding the impact of food on estrogen levels, intestinal microbiota and inflammatory processes is crucial to prevent the development of pathophysiological mechanisms associated with endometriosis. Supplementing the patient’s diet with anti-inflammatory omega 3 is required. Personalized nutritional counseling for individuals with endometriosis may be especially beneficial and necessary, as no single elimination diet can be universally recommended for all patients. An anti-inflammatory diet should be the initial step, followed by more detailed allergy and intolerance screening. 

## Data Availability

Data sharing is not applicable.

## Abbreviations

ACM	allergic contact mucositis
BMI	Body mass index
DHA	docosahexaenoic acid
EPA	eicosapentaenoic acid
FODMAP	fermentable oligo-, di-, monosaccharides and polyols
IBS	irritable bowel syndrome
IGF-1	insulin-like growth factor 1
MUFA	monounsaturated fatty acids
PRISMA	preferred reporting Items for systematic reviews and meta-analyses
PUFA	polyunsaturated fatty acids
SFA	saturated fatty acids
